# Rod Inputs Arrive at Horizontal Cell Somas in Mouse Retina Solely via Rod–Cone Coupling

**DOI:** 10.1523/ENEURO.0427-24.2025

**Published:** 2025-06-03

**Authors:** Wallace B. Thoreson, Asia L. Sladek, Cody L. Barta, Lou E. Townsend

**Affiliations:** ^1^ Truhlsen Eye Institute and Department of Ophthalmology and Visual Sciences; ^2^Department of Pharmacology and Experimental Neuroscience, University of Nebraska Medical Center, Omaha, Nebraska 68106

**Keywords:** cone photoreceptor cells, horizontal cells, optogenetics, retina, rod photoreceptor cells, rod–cone interactions

## Abstract

Rod and cone photoreceptor cells selectively contact different compartments of axon-bearing retinal horizontal cells in the mammalian retina. Cones synapse exclusively on the soma whereas rods synapse exclusively on a large axon terminal compartment. The possibility that rod signals can travel down the axon from terminal to soma has been proposed as a means of producing spectrally opponent interactions between rods and cones, but there is conflicting data about whether this actually occurs. The spectral overlap between rods and cones in mouse makes it difficult to stimulate rod and cone pigments separately. We therefore used optogenetic techniques to analyze photoreceptor inputs into horizontal somas by selectively expressing channelrhodopsin in rods and/or cones. Optogenetic stimulation of rods and cones both evoked large fast inward currents in horizontal cell somas. Cone-driven responses were abolished by eliminating synaptic release in a cone-specific knock-out of the exocytotic calcium sensor, synaptotagmin 1 (Syt1). However, rod-driven responses in horizontal somas were unchanged after eliminating synaptic release from rods but abolished by eliminating release from both rods and cones. This suggests that release from cones is required for transmission of rod signals to horizontal cell somas. Rods and cones are coupled by Cx36 gap junctions, and we found that selective elimination of Cx36 from rods also abolished rod-driven optogenetic responses in horizontal cell somas. Together, these results show that rod signals reach the somas of B-type horizontal cells exclusively via gap junctions with cones and not by transmission down the axon from the axon terminal.

## Significance Statement

Rods and cones contact different compartments of axon-bearing horizontal cells in mammalian retina: cones exclusively contact the soma whereas rods exclusively contact the axon terminal. While cone signals can traverse the axon from soma to terminal, our results show that rod signals cannot travel the other direction. The ability of rod signals to travel from terminal to soma has been proposed as a mechanism for allowing inhibitory interactions between rods and cones. This finding eliminates this pathway as an explanation for opponent rod–cone interactions in color and contrast perception.

## Introduction

Rod and cone photoreceptor cells transduce light into sensory receptor potentials and encode this information into patterns of synaptic vesicle release events (Moser et al., 2020; Thoreson, 2021). Release from photoreceptors is shaped at the first visual synapse by lateral inhibition from second-order horizontal cells. Lateral inhibitory feedback from horizontal cells establishes center-surround receptive fields that enhance the detection of intensity gradients and provides a key substrate for color opponent interactions between different photoreceptors (Thoreson and Mangel, 2012). Rods and cones both form synapses with horizontal cells, and the possibility that rod-driven inputs into horizontal cells provide inhibitory feedback to cones has been invoked to explain several rod–cone interactions. This includes the presence of rod inputs in the inhibitory surround for JAMB OFF-center cells (Joesch and Meister, 2016), color opponency among retinal ganglion cells in ventral regions of mouse retina (Szatko et al., 2020), and suppressive rod–cone interactions (Frumkes and Eysteinsson, 1988; Eysteinsson and Frumkes, 1989).

**Figure 1.**
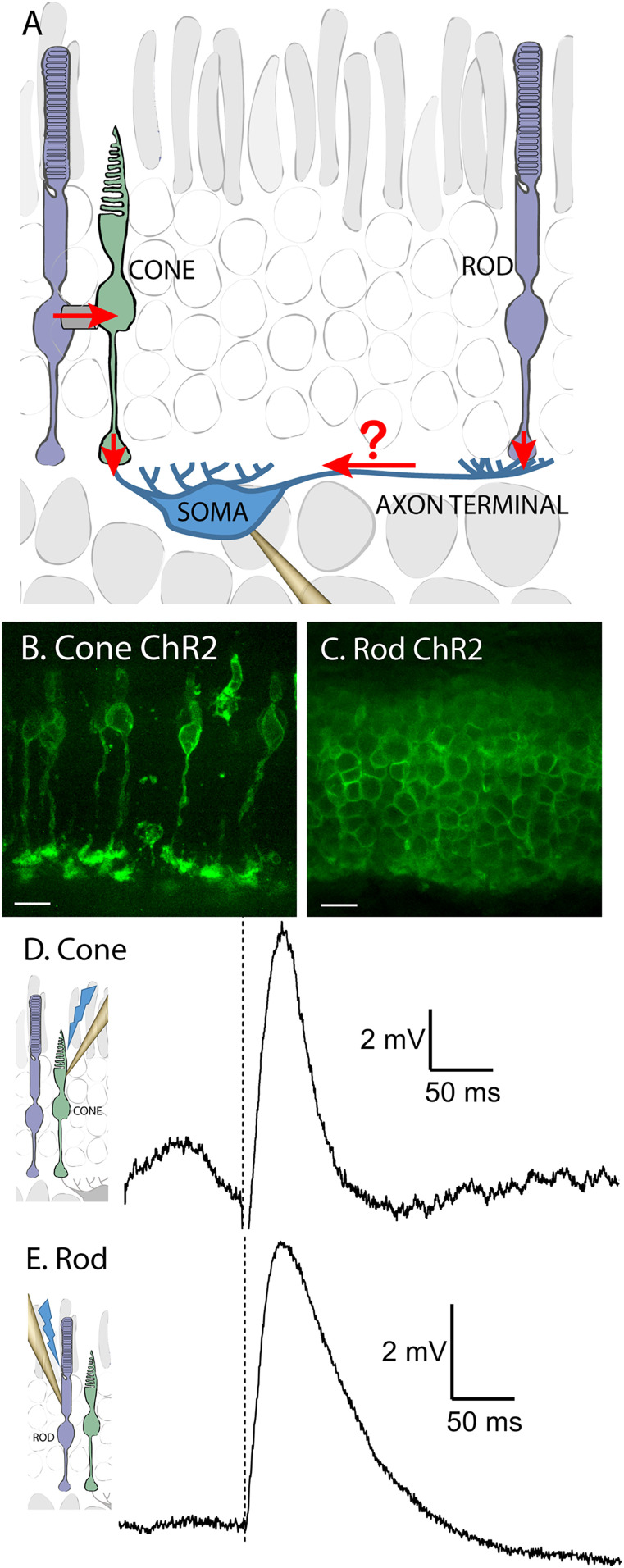
Selective optogenetic stimulation of rods and cones was used to analyze the circuits providing rod input into horizontal somas. ***A***, Diagram illustrating the possible pathways for rod input into B-type, axon-bearing horizontal cells. Rods make contact only with the axon terminal compartment while cones make exclusive contact with the soma. Rod light response can be transmitted via Cx36 gap junctions into cones and from there transmitted to horizontal cell somas via cone synapses. This study addresses the question of whether rod responses can travel from the axon terminal compartment to the soma via the axon. ***B***, Confocal stack showing expression of ChR2/EYFP fusion protein in the membranes of living cones driven by cre-recombinase expression in an HRGP-Cre mouse. ***C***, Confocal stack showing ChR2/EYFP expression in rods driven by Rho-iCre. ***D***, Representative voltage response of a cone expressing ChR2 to 490 nm stimulation (dashed line). ***E***, Example voltage response of a rod expressing ChR2 to optogenetic stimulation (dashed line).

Most mammals have two types of horizontal cells: axonless A-type and axon-bearing B-type cells. In many species, axonless horizontal cells receive preferential or exclusive inputs from S cones (Dacey et al., 1996; Thoreson and Dacey, 2019). Mice have only axon-bearing horizontal cells (Peichl and Gonzalez-Soriano, 1994). As illustrated by the diagram in Figure 1*A*, axon-bearing B-type horizontal cells have two anatomically distinct compartments: the soma and a large dendritic arbor at the axon terminal. In this cell type, cones exclusively contact the soma while rods exclusively contact the axon terminal (Kolb, 1970, 1974; Fig. 1*A*). Rod signals can enter cones via gap junctions and then be transmitted to horizontal cell somas at cone synapses (Fig. 1*A*). Cone-driven light responses are thus readily detected in horizontal cell somas but can also be detected in axon terminals, even in the absence of rod–cone gap junctions, showing that cone signals can travel through the thin axon from soma to terminal (Steinberg, 1969; Nelson et al., 1975; Trumpler et al., 2008). However, it is not clear if rod signals can travel via the axon in the other direction—from terminal to soma.

Rod signals that enter cones via gap junctions necessarily mix with cone signals prior to transmission to horizontal cells. If this were the only pathway available to rods for input into horizontal cell somas, the rod–cone balance of inputs into the soma would necessarily match the rod–cone balance of inhibitory feedback onto cone terminals. However, if rod signals can also enter horizontal cell somas by traveling down the axon from terminal to soma, then responses of horizontal cell somas would differ in their rod–cone balance from cone inputs. This would allow opponent interactions between rods and cones mediated by inhibitory feedback from horizontal cells to cones. Evidence that mouse cones may receive inhibitory feedback from rods thus supported the idea that rod signals might travel from axon terminal to soma (Szikra et al., 2014). On the other hand, other investigators failed to detect any rod-driven responses in horizontal cell somas when gap junctions between rods and cones were eliminated in Cx36KO mice (Trumpler et al., 2008).

Given these conflicting results and the proposed role of this pathway in various visual mechanisms, we directly tested whether rod signals can travel down the axon from terminal to soma in mouse horizontal cells. To do so, we expressed channelrhodopsin2 (ChR2) in rods or cones and recorded optogenetically evoked responses in horizontal cell somas. We compared rod versus cone-driven responses after genetically abolishing synaptic output from one or both photoreceptor cell types. We also tested optogenetic stimulation of rods that lack rod–cone gap junctions. Our results showed that rod inputs enter horizontal cell somas exclusively by traveling through gap junctions to cones and are not transmitted through the axon from terminal to soma. This finding limits the mechanisms available for explaining rod–cone opponent interactions in the retina and visual system.

## Materials and Methods

### Mice

Mice were kept on 12 h dark/light cycles. Mice aged 4–12 weeks of both sexes were used for experiments. Ai32 mice that express channelrhodopsin2 (ChR2)/EYFP fusion protein in the presence of cre-recombinase were obtained from Jackson Labs. Rho-iCre (RRID:IMSR_JAX:015850) mice were also obtained from Jackson Labs (Li et al., 2005; Madisen et al., 2010). Details of HRGP-Cre and Syt1^flox^ (Syt1: MGI:99667) mice have been described previously (Le et al., 2004; Quadros et al., 2017a). Syt1 was selectively eliminated from rods by crossing Syt1^fl/fl^ mice with Rho-iCre/Ai32 mice and eliminated from cones by crossing Syt1^fl/fl^ mice with HRGP-Cre/Ai32 mice. Transgene expression was confirmed by PCR. EYFP, Cre, and iCre PCR was performed by Transnetyx. PCR of Syt1^flox^ and Cx36^flox^ genes was performed in-house using primers described previously (Quadros et al., 2017b; Yao et al., 2018; Grassmeyer et al., 2019). To eliminate Cx36, we originally tried breeding homozygous Cx36 global knock-out mice (Deans et al., 2002) but learned after many fruitless attempts that homozygous Cx36KO mice are infertile. We next obtained Cx36^flox^ mice that allowed selective elimination of Cx36 gap junctions in rods by crossing Cx36^fl/fl^ mice with Rho-iCre/Ai32 mice (Yao et al., 2018; Jin et al., 2020, 2022).

Killing was conducted in accordance with AVMA Guidelines for the Euthanasia of Animals by CO_2_ asphyxiation followed by cervical dislocation. Animal care and handling protocols were approved by the Institutional Animal Care and Use Committee.

### Electrophysiology

Mice were dark-adapted overnight and killed in mid-morning. After killing, retinas were isolated and horizontal slices of retina were prepared as described in detail previously (Feigenspan and Babai, 2017). Briefly, retinas were embedded in 1.8% low temperature gelling agarose (Sigma-Aldrich) and submerged in Ames’ medium supplemented with 5 mM HEPES. Horizontal slices (180–200 μm thick) were cut parallel to the plane of the retina using a vibratome (Leica Microsystems) at room temperature. Tissue slices were placed in the recording chamber and held in place with a slice anchor.

Tissue was superfused at 1–3 ml/min with Ames supplemented with 5 mM HEPES and bubbled with 95% O_2_/ 5% CO_2_. The presence of HEPES limited effects of horizontal cell feedback during these recordings (Hirasawa and Kaneko, 2003). Whole-cell recordings were obtained on an upright fixed-stage microscope (Olympus BX51 or Nikon E600FN) under a water-immersion objective (40× or 60×). We fabricated recording electrodes from borosilicate glass pipettes (1.2 mm OD, 0.95 mm ID, World Precision Instruments) to produce electrodes with tip resistances of 10–12 MΩ. Electrodes were filled with potassium-based pipette solutions containing the following (in mM): 120 KSCN or K-gluconate, 10 TEACl, 10 HEPES, 5–10 EGTA, 1 CaCl_2_, 1 MgCl_2_, 0.5 NaGTP, 5 MgATP, 5 phosphocreatine, 0.01 Alexa 488, pH 7.2–7.3. All chemical reagents were obtained from Sigma-Aldrich unless otherwise indicated.

Perforated patch whole-cell recordings were obtained using either gramicidin or β-escin as the perforating agent. When using gramicidin, we dissolved 5 mg of gramicidin into 1 ml of 95% ethanol on the day of the experiment. We then dissolved 0.5 μl of this stock solution into 0.5 ml of the pipette solution for a final concentration of 5 μg/ml (Kyrozis and Reichling, 1995). When using β-escin, we prepared a stock solution on the day of an experiment by dissolving 1.4 mg of β-escin into 0.1 ml pipette solution. We then transferred 1 ml of this stock solution into 0.5 ml of the pipette solution for a final concentration of 25 μM. β-Escin powder needs to be stored under dark, dry conditions to prevent transformation into inactive α-escin (Sarantopoulos et al., 2004).

Horizontal cells were identified visually and confirmed physiologically by their characteristic voltage-dependent currents, particularly prominent A-type K^+^ currents (Feigenspan and Babai, 2015). Horizontal cell identity was further confirmed in some cases by loading cells with the fluorescent dye Alexa 488 through the patch pipette.

Recordings were performed in voltage clamp using an Axopatch 200B (Axon Instruments/Molecular Devices) with AxoGraph X or pClamp10 acquisition software and digitized with an ITC-18 interface (Heka Instruments) or Digidata 1550 (Molecular Devices). Membrane currents were acquired at 10 kHz sampling and filtered at 1 kHz. Voltages were not corrected for liquid junction potentials (gluconate pipette solution, 12 mV; KSCN pipette solution, 3.9 mV). Membrane capacitance, membrane resistance, and access resistance in horizontal cells averaged 16.9 ± 8.9 pF, 305.4 ± 252.1 MΩ, and 19.0 ± 4.7 MΩ (*n* = 41).

ChR2 was activated by a 1–10 ms pulse of 490 nm light from an LED (Lambda TLED, Sutter Instrument). The voltage was regulated using a computer-controlled analog input to the LED. For experiments reported here, we used a voltage of 4 V to generate consistently saturating responses.

### Immunohistochemistry

For immunohistochemical experiments, eyes were enucleated immediately after killing and then placed in oxygenated Ames’ medium. After removing the cornea and lens, the posterior eyecup was fixed in 4% paraformaldehyde for 40 min., washed in PBS three times for 10 min each, and cryoprotected in 30% sucrose overnight at 4°C. Eyecups were then embedded in OCT compound (Sakura Finetek USA) and stored at −80°C until sectioning at 25 µm with a cryostat (Leica CM 1800). Retinal sections were treated with a blocking solution of 6% goat serum (Jackson ImmunoResearch and Life Technologies, respectively) for 1 h at room temperature before applying the primary antibody. For Syt1, we used a primary camelid antibody targeting the cytoplasmic tail and conjugated to Atto-488 (Synaptic Systems, N2302-At488-L; 1:500). To target Cx36, we used a mouse monoclonal antibody targeting the loop region (1E5H5; Invitrogen, 37–4600; 1:400) visualized with a rhodamine Red-X secondary antibody (goat anti-mouse IgG; Invitrogen, R-6393; 1:200). Primary and secondary antibodies were diluted to working concentrations in blocking solution. Sections were incubated in primary antibody at 4°C overnight and washed in 1× PBS six times for 10 min each the following morning. Secondary antibody was applied at room temperature for 1 h. Retinal sections were covered with standard coverslips and mounted with Vectashield (VectorLabs, RRID:AB_2336787) before imaging.

### Imaging

Confocal images were obtained using Nikon Elements software and a laser confocal scanhead (PerkinElmer Ultraview LCI) equipped with a cooled CCD camera (Hamamatsu Orca ER) mounted on a Nikon E600FN microscope. Fluorescent excitation was delivered from an argon/krypton laser at 488 or 568 nm wavelengths with emission collected at 525 or 607 nm, respectively. Filters were controlled using a Sutter Lambda 10–2 filter wheel and controller. The height of the objective (60× water immersion, 1.2 NA, Plan Apo with correction collar, Nikon) was controlled using a E662 *z*-axis controller (Physik Instrumente). Images were adjusted for color, brightness, and contrast using Nikon Elements, Fiji/ImageJ, and Adobe Photoshop software.

### Statistical analysis

Statistical analysis and data visualization were performed using GraphPad Prism. Where applicable, *p* values were adjusted for multiple comparisons using Tukey's multiple-comparisons tests along with one-way ANOVA. The criterion for statistical significance was set at *α* = 0.05. Data are presented as mean ± SD.

## Results

To compare rod and cone inputs into the soma compartment of mouse horizontal cell, we selectively expressed ChR2 in rods and/or cones. To do so, we crossed Rho-iCre and HRGP-Cre mice that express Cre-recombinase in rods and cones, respectively, with Ai32 mice that express a Cre-driven channelrhodopsin-2 (ChR2)/EYFP fusion protein. Figure 1*B* shows membrane expression of ChR2/EYFP fusion protein in live cones driven by cre-recombinase expression in an HRGP-Cre mouse. Figure 1*C* shows ChR2/EYFP expression in live rods driven by Rho-iCre. With these mice, we could use blue light to optogenetically stimulate either rods or cones.

Using perforated patch recording techniques with gramicidin or β-escin as a perforating agent, we found that optogenetic stimulation evoked depolarizing voltage changes in rods of 7.0 ± 3.54 mV (*n* = 11) and cones of 6.6 ± 3.67 mV (*n* = 4). Representative responses from a cone and rod are shown in Figure 1*D* and *E*, respectively. Rod voltage responses rose with a time constant of 8.8 ± 2.5 ms and reached their peak after 26.0 ± 13.6 ms. Cone responses were similar, attaining a peak response after 19.6 ± 8.8 ms.

**Figure 2.**
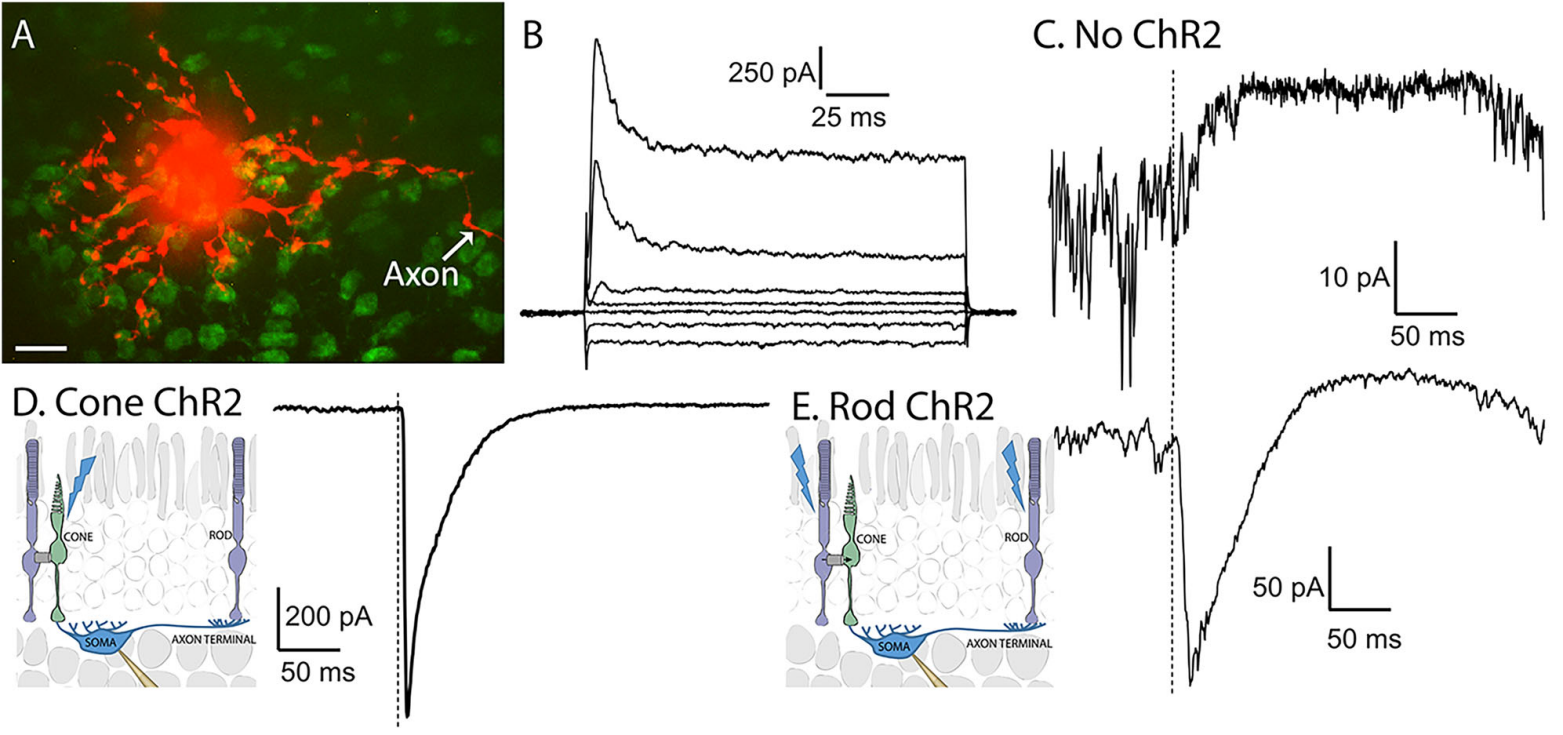
Responses of horizontal cell somas to optogenetic stimulation of rods and cones. ***A***, Confocal image of a horizontal cell (red) filled with sulforhodamine B introduced through a patch pipette during whole-cell recording. The initial portion of the axon is visible but the more distant terminal compartment is not. Cone terminals were labeled by bath application of FITC-conjugated peanut agglutinin (green). Scale bar, 10 µm. ***B***, Example of currents evoked by a series of voltage steps (20 mV steps, from −100 to +20 mV) applied to a voltage-clamped horizontal cell (held between steps at −60 mV). Rapidly inactivating A-type outward currents are a characteristic of horizontal cells. ***C***, Example response of a horizontal cell soma to a 1 ms 490 nm flash applied to a control C57Bl6J retina without ChR2. By activating endogenous cone opsins, the bright blue light used for optogenetic stimulation can hyperpolarize cones, thereby reducing ongoing glutamate release and resulting in an outward current in postsynaptic horizontal cells. ***D***, Example of the fast inward current evoked in a horizontal cell soma by optogenetic activation of cones expressing ChR2. ***E***, Example response of a horizontal cell soma to optogenetic activation of rods expressing ChR2. Diagrams in ***D*** and ***E*** illustrate the recording arrangements in these experiments.

To record from horizontal cell somas, we prepared horizontal slices of retina, slicing along the plane of the inner nuclear layer to expose horizontal cells while retaining intact rods and cones (Feigenspan and Babai, 2017). Figure 2*A* shows a confocal image of a horizontal cell soma (red) filled with sulforhodamine B introduced through a patch pipette during whole-cell recording. The initial segment of the horizontal cell axon is also visible (arrow) although the more distant axon terminal is not. Cone terminals in this image were labeled with FITC-conjugated peanut agglutinin (green). In addition to their characteristic morphology, horizontal cells could be identified from a characteristic set of voltage-dependent currents that included prominent A-type outward K^+^ currents (Fig. 2*B*; Feigenspan and Babai, 2015). In recordings from horizontal cells in retinas that lacked ChR2 but retained intact Syt1 in cones, the bright blue light used for optogenetic stimulation often evoked slow outward currents (Fig. 2*C*). These currents arose from the activation of endogenous S cone opsins in these light-adapted retinas. Light-evoked activation of endogenous opsins causes cones to hyperpolarize which in turn leads to diminished glutamate release, producing outward currents in postsynaptic horizontal cells. In contrast with the small, slow outward currents that accompanied activation of endogenous cone opsins, optogenetic stimulation of cones and rods that expressed ChR2 evoked large, fast inward currents in horizontal cell somas (Fig. 2*D*,*E*). Endogenous opsins also sometimes contributed a late-developing outward current (Fig. 2*E*).

**Figure 3.**
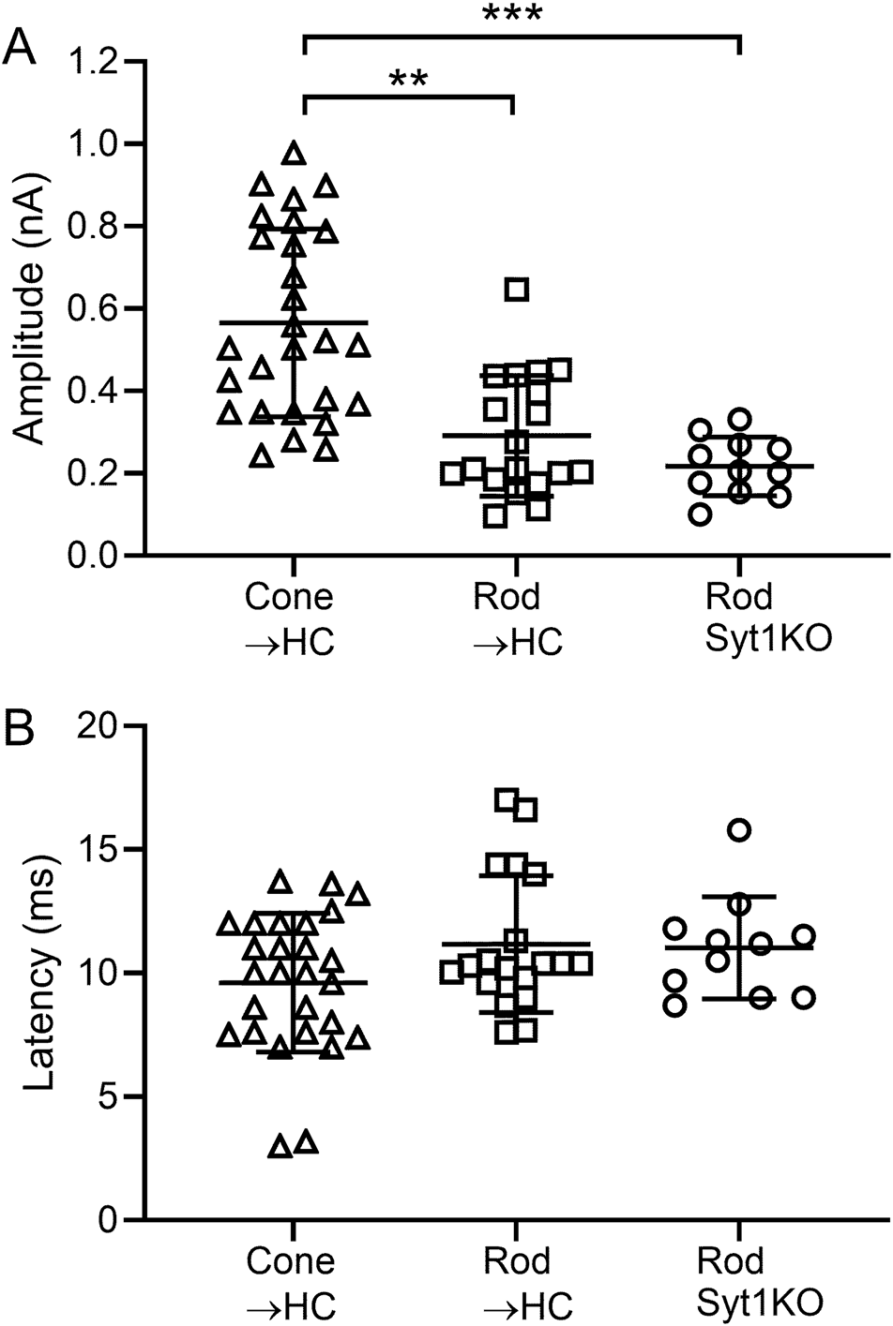
Summary data of current responses in horizontal cell somas evoked by optogenetic activation of cones (Cone → HC) and rods (Rod→HC). Data are also plotted for horizontal cell responses to optogenetic activation of rods that lacked Syt1 (Rod Syt1KO; Rho iCre/Syt1^fl/fl^). ***A***, Peak amplitude of the inward optogenetically evoked current (±SD). Cone-driven: 565 ± 228 pA, *n* = 27. Rod-driven: 291 ± 228 pA, *n* = 19. Rod-driven/rod-specific Syt1KO: 217 ± 71.3 pA, *n* = 11. ***, cone-driven versus rod-driven; *p* < 0.0001, cone-driven versus rod-driven/rod-specific Syt1KO: *p* < 0.0001, Tukey's multiple-comparisons test. ***B***, Latency to the peak inward current did not differ among the three conditions. Cone-driven: 9.6 ± 2.80 ms. Rod-driven: 11.2 ± 2.76 ms. Rod-driven/rod-specific Syt1KO: 11.0 + 2.06 ms.

Figure 3 summarizes differences in amplitude and latency of rod- and cone-driven inward optogenetic currents in horizontal cell somas. The peak amplitude of the inward optogenetically evoked current in horizontal cell somas was larger when driven by optogenetic stimulation of cones (565 ± 228 pA; *n* = 27) than rods (291 ± 228 pA; *p* < 0.0001; *n* = 19). The latency to the peak of the inward current did not differ significantly between cone-driven (9.6 ± 2.80 ms; *n* = 27) and rod-driven (11.2 ± 2.76 ms; *n* = 19) responses. The peak of the optogenetically evoked inward horizontal cell current was achieved before the peak of the optogenetically evoked depolarizing voltage response in photoreceptors showing that synaptic release was efficiently triggered during the initial depolarization of these cells.

To analyze pathways by which rod signals enter horizontal cell somas, we genetically eliminated the Ca^2+^ sensors controlling release in rods or cones. The presynaptic Ca^2+^ sensor Syt1 controls fast glutamate release from both rods and cones (Grassmeyer et al., 2019; Mesnard et al., 2022). We eliminated this Ca^2+^ sensor selectively from rods and/or cones by crossing Syt1^fl/fl^ mice with Ai32 mice that express ChR2 and then crossing their offspring with HRGP-Cre and/or Rho-iCre mice to drive cre-recombinase in cones, rods, or both. Selective elimination of Syt1 from cones or rods in homozygous Syt1^fl/fl^ mice by coexpression of HRGP-Cre or Rho-iCre, including deletion of Syt1 from ChR2-expressing mice, was confirmed in earlier studies (Grassmeyer et al., 2019; Mesnard et al., 2022; Sladek and Thoreson, 2023).

**Figure 4.**
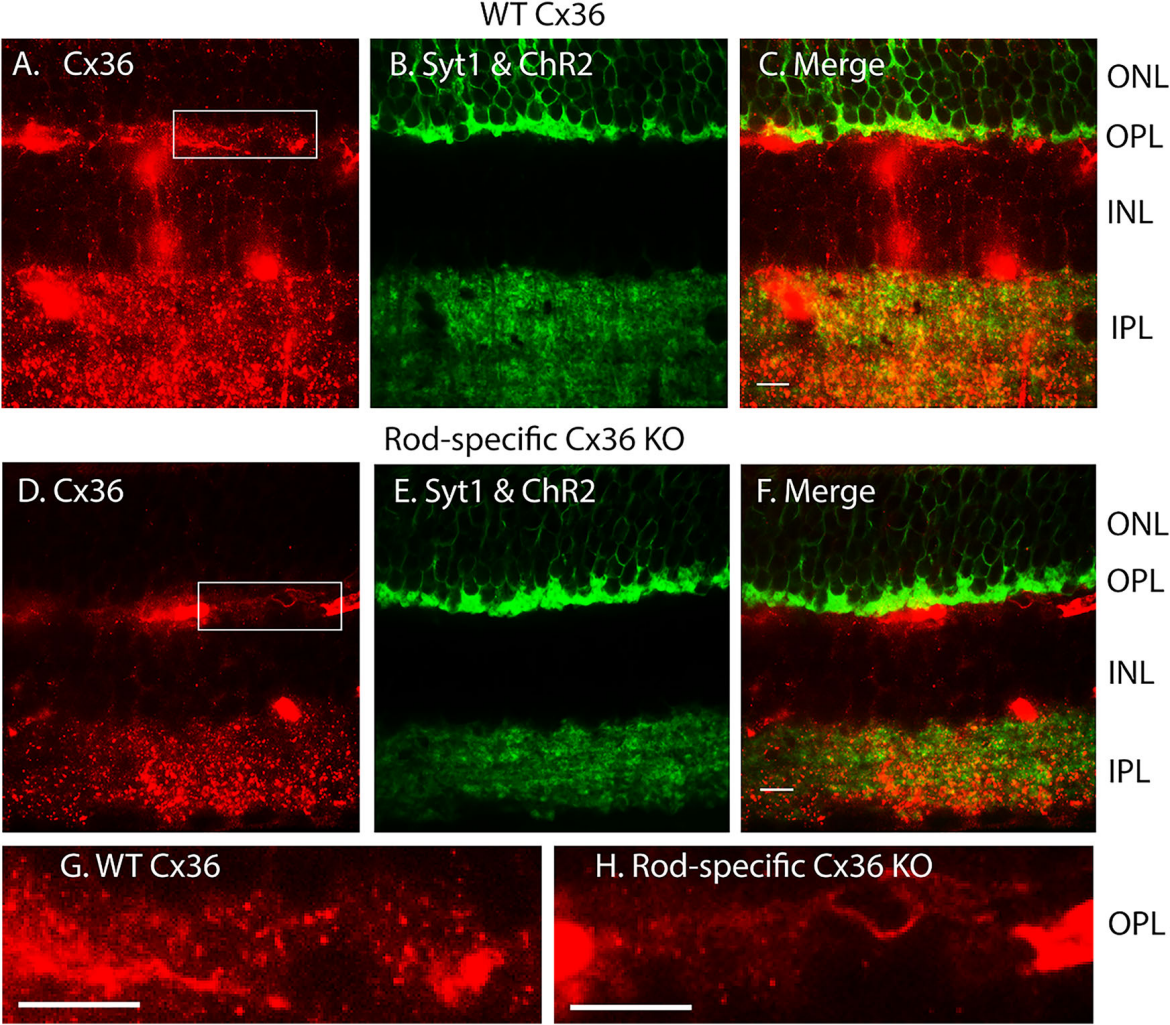
Selective elimination of Cx36 from rods in rod-specific Cx36 knock-out (KO) mice was confirmed by immunohistochemistry. Top panel shows single confocal sections from an Ai32/Rho-iCre mouse expressing wild-type Cx36. ***A***, Immunolabeling for Cx36 using a rhodamine-conjugated secondary antibody. Blood vessels are also strongly labeled by rhodamine. ***B***, Green fluorescence in the outer nuclear layer (ONL) is due to expression of EYFP/ChR2 fusion protein in rods. There is additional strong immunofluorescence in the outer plexiform layer (OPL) from Syt1 labeled with an Atto488-tagged nanobody. Cx36 puncta were evident in both the OPL and inner plexiform layer (IPL). Syt1 fluorescence was prominent at synapses in the OPL and IPL, but not the inner nuclear layer (INL). ***C***, Merged image showing overlaid red and green fluorescent images. Middle row of figures shows single confocal sections from an Ai32/Rho-iCre/Cx36^flfl^ mouse (rod-specific Cx36 KO). ***D***, Immunolabeling for Cx36 using a rhodamine-conjugated secondary antibody. While puncta in the IPL remain strongly labeled, Cx36 puncta are largely absent from the OPL. ***E***, Syt1 and ChR2/EYFP fluorescence. ***F***, Merged image. ***G***, Magnified image of a capillary-free region of the OPL showing Cx36 immunofluorescent puncta in the Ai32/Rho-iCre confocal section from panel ***A*** (region enclosed by the box). ***H***, Magnified image of a capillary-free region of the OPL illustrating the absence of Cx36 immunofluorescent puncta in the rod-specific Cx36 KO mouse section shown in panel ***D*** (region enclosed by the box).

To eliminate rod input into cones via gap junctions, we genetically eliminated Cx36 from rods by crossing Ai32 mice with mice that express Rho-iCre and Cx36^flfl^ genes (Yao et al., 2018; Jin et al., 2020, 2022). To confirm that Cx36 was selectively eliminated from rods in these mice, we compared Cx36 labeling in Ai32/Rho-iCre mice (wild-type Cx36) with Ai32/Rho-iCre/Cx36^flfl^ (rod-specific Cx36 KO). EYFP/ChR2 expression in rods produced green fluorescence in the ONL, even in fixed tissue (Fig. 4). In this experiment, we also labeled Syt1 with an Atto488-tagged camelid antibody. Along with the existing EYFP fluorescence, this produced strong labeling of synapses in both the IPL and OPL (Fig. 4). Because of the EYFP fluorescence, we used a red fluorophore, rhodamine, to label Cx36, despite the fact that this dye also labels blood vessels quite readily. In capillary-free regions of the OPL from a mouse that expressed wild-type Cx36, one can see punctate labeling of the Cx36 antibody, similar to that described previously (Jin et al., 2020). As illustrated by the confocal sections in Figure 4, this punctate labeling was largely eliminated in rod-specific Cx36KO mice, while staining for Cx36 puncta in the IPL was unchanged. Although cones can still express Cx36 in these mice, elimination of Cx36 from rods is sufficient to eliminate functional gap junctions between rods and cones (Jin et al., 2020, 2022). The few remaining puncta in the OPL may represent labeling of Cx36 in cone terminals (Jin et al., 2020). Elimination of puncta in the OPL can be seen more easily in the magnified image at the bottom of Figure 4. Labeling for Syt1 was not altered in rod-specific Cx36KO mice.

**Figure 5.**
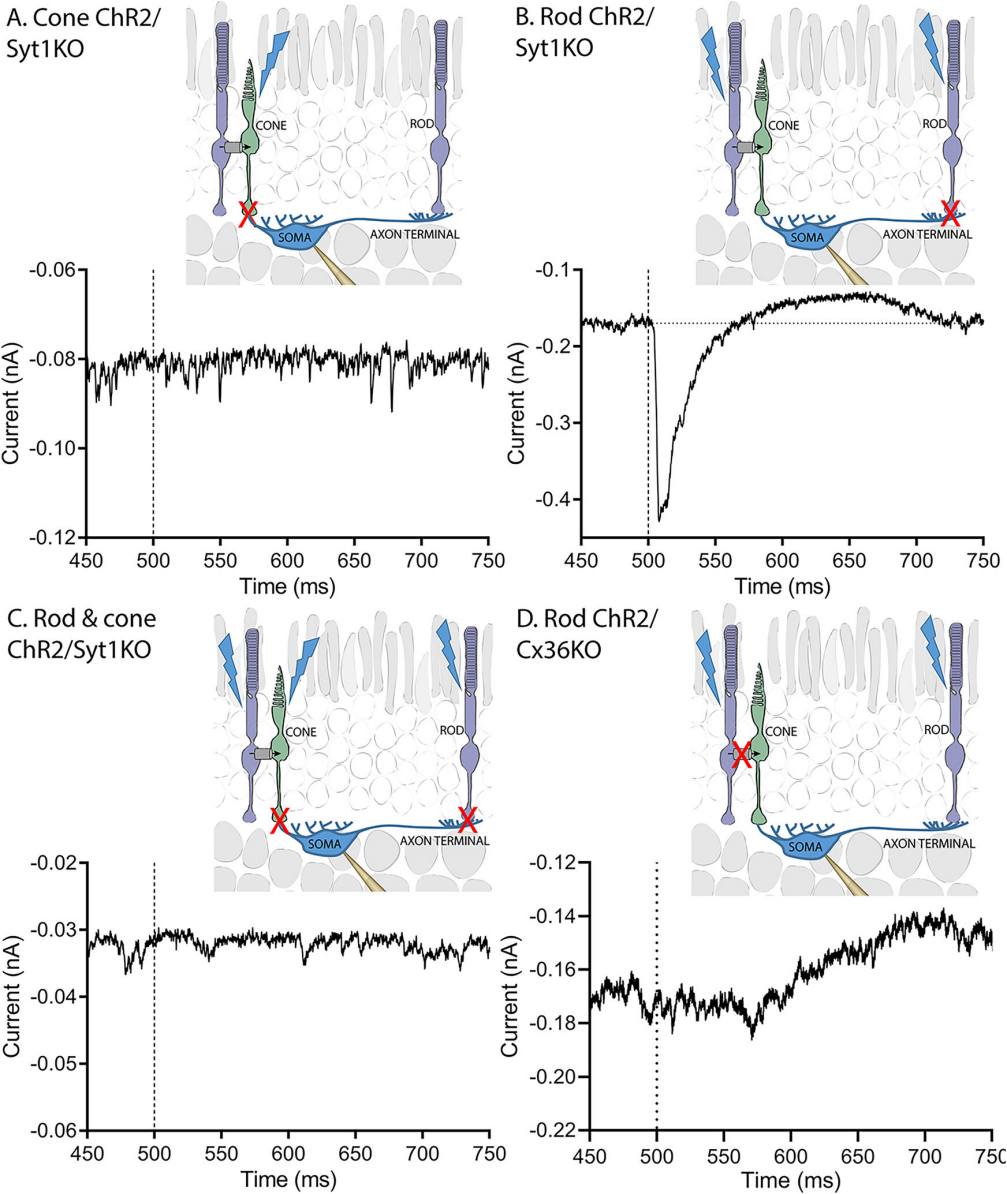
Rod signals enter horizontal somas exclusively through gap junctions with cones. ***A***, Example from a horizontal cell soma showing that selective elimination of Syt1 from cones (Syt1^fl/fl^ × HRGP-Cre × Ai32) abolished the response to optogenetic stimulation of cones (compare with Fig. 1*E*). ***B***, Example horizontal cell response showing that rods remained able to evoke large optogenetic responses in the absence of Syt1 (rod-specific Syt1KO: Syt1^fl/fl^ × Rho-iCre × Ai32). ***C***, Example horizontal cell response showing that eliminating Syt1 from both rods and cones (Syt1^fl/fl^ × HRGP-Cre × Rho-iCre × Ai32) eliminated responses to simultaneous optogenetic stimulation of both photoreceptors. ***D***, Example response showing that elimination of gap junctions between rods and cones produced by removal of Cx36 from rods (Cx36^fl/fl^ × Ai32 × Rho-iCre) abolished responses in horizontal cell somas evoked by optogenetic stimulation of rods. The slow, delayed outward currents in panels ***B*** and ***D*** are due to activation of endogenous cone opsins and the resulting decrease in glutamate release from cones that retain functional Syt1.

Figure 5 shows representative horizontal cell responses to optogenetic stimulation of rods and/or cones following selective elimination of Syt1 from cones (A), rods (B), or both rods and cones (C). Figure 5 also illustrates the optogenetic response produced after selective elimination of Cx36 from rods (D). The sites targeted by these genetic manipulations are illustrated diagrammatically.

As illustrated in Figure 5*A*, selectively eliminating Syt1 from cones abolished horizontal cell responses to optogenetic stimulation of cones. Optogenetic responses were absent in all seven horizontal cells recorded in three cone-specific Syt1 knock-out retinas. These results support the conclusion that cone responses in the horizontal cell soma compartment require direct synaptic input from cones. Any cone responses that might enter rods via gap junctions do not appear capable of reaching the horizontal cell soma. While evoked responses were abolished, spontaneous miniature excitatory postsynaptic currents (mEPSCs) persisted in horizontal cells after loss of Syt1 in cones, consistent with results obtained previously by recording glutamate transporter currents in cones (Fig. 5*A*; Grassmeyer et al., 2019). The presence of mEPSCs shows that the cell was healthy and synapses remained functional.

As illustrated in Figure 5*B*, optogenetic stimulation of rods lacking Syt1 still produced large inward currents in horizontal cell somas. mEPSCs were also present. This contrasts with absence of detectable responses in horizontal cells following optogenetic stimulation of cones that lacked Syt1. As summarized by the graphs in Figure 3, eliminating synaptic transmission from rods by genetically eliminating Syt1 did not significantly reduce the amplitude of inward currents evoked by optogenetic stimulation of rods. The latency to peak current was also no different in mice with and without Syt1 in rods (Fig. 3).

We next eliminated Syt1 from both rods and cones. To do so, we crossed Syt1^fl/fl^ mice with Ai32 mice that express ChR2 as well as HRGP-Cre and Rho-iCre mice that express cre-recombinase in cones and rods, respectively (Syt1^fl/fl^ × HRGP-Cre × Rho-iCre × Ai32). Since ChR2 is expressed in both rods and cones, optogenetic stimulation activates both cell types. However, unlike the situation when synaptic transmission was eliminated only from rods, the added loss of transmission from cones abolished horizontal cell responses to optogenetic stimulation (Fig. 5*C*; 5 cells from 3 mice). Once again, spontaneous Ca^2+^-independent release of vesicles persisted in the absence of Syt1 showing that cells remained healthy and synapses were intact (Fig. 5*C*). These data show that the entry of rod-driven responses into horizontal cell somas requires intact cone release, suggesting that rod input instead reaches horizontal cell somas by transmission into cones via gap junctions.

To test the requirement for rod/cone gap junctions, we recorded from mice in which Cx36 was selectively eliminated from rods while Syt1 remained (Cx36^fl/fl^ × Ai32 × Rho-iCre). As mentioned earlier, eliminating Cx36 from rods abolishes functional gap junctions between rods and cones. In rod-specific Cx36KO mice, responses evoked by optogenetic stimulation of rods cannot be transmitted to cones via gap junctions and thus can only enter horizontal somas by transmission down the axon from rod input into the axon terminal compartment. Horizontal cells in these retinas exhibited mEPSCs and outward cone-driven light responses to the blue LED showing that they retained functional synaptic input from cones (Fig. 5*D*). However, as illustrated by the example in Figure 5*D*, optogenetic stimulation of rods lacking gap junctions with cones failed to evoke inward currents in horizontal cell somas (Fig. 5*D*; *n* = 8 cells from 3 mice) showing that rod–cone gap junctions are also required for transmission of rod responses to horizontal cell somas.

## Discussion

The results of this study show that the entry of rod signals into horizontal cell somas requires both rod–cone gap junctions and intact synaptic output from cones. While rod signals can enter horizontal cell somas by traveling into cones via gap junctions from rods, they were abolished in the absence of rod–cone gap junctions and by eliminating Syt1 from cones. Our data thus support earlier suggestions (Trumpler et al., 2008) that cone signals can pass down the axon into the rod-driven horizontal cell terminal compartment, but rod signals cannot travel the other direction—from axon terminal to soma. The spectral sensitivity of horizontal cell somas therefore necessarily matches the spectral sensitivity of the cone input signal. Without a spectral difference, feedback from horizontal cell dendrites to cone somas cannot produce spectral opponency. The responses seen in earlier recordings that suggested the presence of rod to cone feedback were quite small, raising the possibility that field potentials present in outer retina may have confounded interpretation (Szikra et al., 2014).

The asymmetry that allows cone signals to travel from soma to terminal but prevents rod signals from traveling the other direction involves asymmetries in input resistance, with a much higher input resistance in the axon terminal (Nelson et al., 1975; Yagi and Kaneko, 1988; Golard et al., 1992). Although postsynaptic currents will be attenuated by passage down the fine axon, small currents that reach the high resistance terminal compartment can generate measurable voltage changes. This is particularly evident in certain fish horizontal cells where the axon terminal compartment does not receive any direct photoreceptor input but nevertheless shows cone-driven light responses (Stell, 1975; Weiler and Zettler, 1979).

Responses of rods and cones interact at many levels of the visual system. The earliest site involves gap junctions between rods and cones (Deans et al., 2002; Volgyi et al., 2004; Fain and Sampath, 2018; Jin et al., 2022; Sladek and Thoreson, 2023), allowing a pathway for rod signals to enter the cone circuitry. This so-called secondary pathway transmits rod signals more significantly at higher light intensities than the primary rod pathway involving direct contacts between rods and rod ON bipolar cells (Volgyi et al., 2004; Jin et al., 2022). Another early circuit by which rods and cones can interact involves inhibitory feedback from horizontal cells. Similar to cones, rods receive inhibitory feedback from postsynaptic horizontal cells (Thoreson et al., 2008; Babai and Thoreson, 2009). While our data argue against inhibitory feedback from rods to cones at horizontal cell somas, the presence of cone signals in the axon terminal compartment provides a substrate for inhibitory feedback of cone responses to rods. Mouse cones are more sensitive to short wavelengths than rods and so inhibitory feedback of mixed rod/cone signals in horizontal axon terminals to presynaptic rods would allow S/M wavelength opponent interactions. Like the somas of axon-bearing B-type horizontal cells, the axonless A-type horizontal cells found in other mammalian species do not receive rod inputs (Kolb, 1974; Dacey et al., 1996; Pan and Massey, 2007)

The conclusions of this study limit potential sites available for opponent rod/cone interactions. For example, JAMB ganglion cells in mice show a center OFF response to UV light and surround ON response driven by rods (Joesch and Meister, 2016). Pharmacological experiments suggested that the surround response arose from horizontal cell feedback of rod signals to S cones. However, our data show that rod signals cannot travel into the soma via the axon, suggesting this pathway is unlikely to mediate the surround of JAMB cells and so more likely involves inner retinal circuits. Similarly, horizontal cell feedback from rods to cones has been suggested to mediate suppressive rod–cone interactions observed both psychophysically and at the cellular level in amphibians (Frumkes and Eysteinsson, 1988; Eysteinsson and Frumkes, 1989). In suppressive rod–cone interactions, the activity of rods in darkness suppresses cone-driven light responses. While feedback from rods to cones may play a role in amphibians, our data argue that horizontal cell feedback from rods to cones is unlikely to be the mechanism for suppressive rod–cone interactions in mammals.

## Synthesis

Reviewing Editor: Arianna Maffei, Stony Brook University Decisions are customarily a result of the Reviewing Editor and the peer reviewers coming together and discussing their recommendations until a consensus is reached. When revisions are invited, a fact-based synthesis statement explaining their decision and outlining what is needed to prepare a revision will be listed below. The following reviewer(s) agreed to reveal their identity: Steven Barnes, Catherine Morgans.

The reviewers agree that the manuscript provides a valuable contribution to the field and the experiments are well designed and executed.

The reviewers suggested adding a visual diagram of the circuit under study to help readers who are not experts in the field understand the goals of the work.

They also indicated the need for the addition of images showing the validation of the transgenic lines and clarification about the data presented in Figure 3.

Reviewer #1:

This excellently designed set of experiments have managed to bridge a previous gap in the understanding of a critical, early synaptic interaction at the first visual synapse. The blending of ChRh2 and patch electrode herein allowed convincing recordings of a previously unexplorable issue with remarkably clear results. This major component of the manuscript is outstanding. This reviewer tested the theory that the problem being studied was overly confusing to unfamiliar scientifically trained persons in other fields (and who did not provide "assistance with this review" in the normal sense): They got stuck envisioning the unusual 'cell body' and 'axon terminal' nomenclature used by persons that work in the outer retinal. They were unable to envision the organization of the photoreceptor synapses to the two functionally independent but common parts of the horizontal cell. Once explained, they got it and were convinced of the correct nature of the research outcome. The experience told this reviewer that a visual abstract, or an additional explanatory graphic figure, could be used to map-out the axon terminal and the cell body of horizontal cells, showing their common nature but separate signaling roles, such that, now, thanks to the electrophysiology and the used of targeted synaptotagmin 1 expression is easier to visualize without reaching into previous publications.

Reviewer #2:

The authors investigate the response of retinal horizontal cells (HCs) to rod and cone inputs. Cones are known to synapse onto the somato-dendritic region of the HC, whereas rods synapse onto the axon terminal processes. The authors sought to determine the route by which rod inputs might reach the HC soma. The two possible routes are 1) from the rod synaptic terminal to the HC axon terminal by synaptic transmission and then to the HC soma via the HC axon, or 2) from the rod terminal to the cone terminal via cx36 gap junctions and then from the cone terminal to the HC soma by synaptic transmission. To compare the two possibilities, they designed and conducted an elegant set of experiments using a transgenic strategy. They expressed channel rhodopsin in either rods or cones enabling them to stimulate one or the other selectively, and then knocked out synaptic transmission from rods or cones, or knocked out rod-cone gap junctions. The results cleanly demonstrate that rod inputs to the HC are unable to travel to the HC soma via the HC axon, but depend on rod-cone gap junctional coupling. The results have implications for understanding the circuitry underlying opponent rod-cone interactions in color and contrast perception.

The following points need to be addressed for publication:

The perforated patch procedure should be described in the Materials and Methods.

To validate the transgenic genotypes, please show the following images:

Confocal images of YFP fluorescence in the Ai32/Rho-iCre and Ai32/HRGP-Cre should be added to Figure 1 or included as a supplementary figure.

Confocal images of Syt1 immunofluorescence in the OPL of wild type, rod ChR2/Syt1KO, cone ChR2/Syt1KO, and rod/cone ChR2/Syt1KO need to be added to Figure 3.

Confocal images of cx36 immunofluorescence in the OPL of wild type and Rod ChR2/Cx36KO need to be added to Figure 3.

The text referring to Figure 3 in the Results states "Optogenetic responses were absent in all 7 horizontal cells recorded in 3 cone-specific Syt1 knockout retinas" and "(Fig. 3C; 5 cells from 3 mice)" and "(Fig. 3D; n=8 cells from 3 mice)", but the current traces in Figs 3A, 3C, and 3D seem to be from only one cell each. Please make this clear in the figure legend.

Figure 3: The Results refers to the presence of spontaneous miniature post-synaptic currents in Fig 3A and 3C, but not 3D. Why not mention minis in 3D? What is the explanation for the apparent difference in the time course of the minis in 3A and 3D (the ones in 3A appear faster)?

Is there an explanation for the slow decrease in the current in 3D?

## Author Response

Synthesis of Reviews:

Editor: The reviewers agree that the manuscript provides a valuable contribution to the field and the experiments are well designed and executed.

The reviewers suggested adding a visual diagram of the circuit under study to help readers who are not experts in the field understand the goals of the work.

They also indicated the need for the addition of images showing the validation of the transgenic lines and clarification about the data presented in Figure 3.

Author reply: >>We apologize for the delay in returning this paper. It took us a while to breed the mice needed for IHC experiments on Cx36 requested by reviewer 2. We revised the paper to include this additional transgenic validation and a visual diagram of the circuit along with other changes described below. >>In earlier work from our laboratory, we performed an extensive series of studies confirming selective elimination of Syt1 from rods and cones using the floxed Syt1 mouse line that we developed. These earlier studies included showing selective elimination of Syt1 from rods and/or cones in Ai32 mice. We genotyped all of the mice used in the present experiments and are confident that Syt1 was successfully eliminated from rods and cones in the mice used for this study. We also no longer have Syt1fl/fl/Ai32/RhoiCre/HRGPCre mice and the breeding needed to generate these mice originally took over 2 years. Finally, green fluorescence from EYFP (shown in new Fig. 4) obscures detection of changes in the green channel making dual color imaging problematic. >>We completed experiments on Syt1 over a year ago during which time we obtained and bred the necessary Cx36KO mice. Since we had not previously validated the floxed Cx36 mouse line that we obtained from Dr. Ribelayga, we followed the reviewer's suggestions and performed IHC studies to test whether Cx36 was selectively eliminated from rods in Rho-iCre/Ai32/Cx36flfl mice. This characterization took a few months to breed the mice needed for these experiments and refine IHC protocols. These new data form the basis for new Figure 4. >>Finally, we revised the text throughout in an effort to make everything a bit more clear.

Reviewer #1:

This excellently designed set of experiments have managed to bridge a previous gap in the understanding of a critical, early synaptic interaction at the first visual synapse. The blending of ChRh2 and patch electrode herein allowed convincing recordings of a previously unexplorable issue with remarkably clear results. This major component of the manuscript is outstanding. This reviewer tested the theory that the problem being studied was overly confusing to unfamiliar scientifically trained persons in other fields (and who did not provide "assistance with this review" in the normal sense): They got stuck envisioning the unusual 'cell body' and 'axon terminal' nomenclature used by persons that work in the outer retinal. They were unable to envision the organization of the photoreceptor synapses to the two functionally independent but common parts of the horizontal cell. Once explained, they got it and were convinced of the correct nature of the research outcome. The experience told this reviewer that a visual abstract, or an additional explanatory graphic figure, could be used to map-out the axon terminal and the cell body of horizontal cells, showing their common nature but separate signaling roles, such that, now, thanks to the electrophysiology and the used of targeted synaptotagmin 1 expression is easier to visualize without reaching into previous publications.

Authors: >>As suggested, we added a diagram to Fig. 1A illustrating possible rod and cone inputs into horizontal cell somata and axon terminals, placing a question mark over the putative pathway down the axon from terminal to soma. We broke old Fig. 1 into two figures and added diagrams to new Figs. 1 and 2 that illustrate the recording arrangements used for optogenetic stimulation of rods vs. cones. We also re-drew the diagrams that accompany optogenetic response waveforms in Fig. 5 (old Fig 3) to emphasize the distance between soma and terminal and more clearly identify the different sites that were genetically eliminated.

Reviewer #2:

The authors investigate the response of retinal horizontal cells (HCs) to rod and cone inputs. Cones are known to synapse onto the somato-dendritic region of the HC, whereas rods synapse onto the axon terminal processes. The authors sought to determine the route by which rod inputs might reach the HC soma. The two possible routes are 1) from the rod synaptic terminal to the HC axon terminal by synaptic transmission and then to the HC soma via the HC axon, or 2) from the rod terminal to the cone terminal via cx36 gap junctions and then from the cone terminal to the HC soma by synaptic transmission. To compare the two possibilities, they designed and conducted an elegant set of experiments using a transgenic strategy. They expressed channel rhodopsin in either rods or cones enabling them to stimulate one or the other selectively, and then knocked out synaptic transmission from rods or cones, or knocked out rod-cone gap junctions. The results cleanly demonstrate that rod inputs to the HC are unable to travel to the HC soma via the HC axon, but depend on rod-cone gap junctional coupling. The results have implications for understanding the circuitry underlying opponent rod-cone interactions in color and contrast perception.

The following points need to be addressed for publication:

The perforated patch procedure should be described in the Materials and Methods.

Authors: >>As requested, we added a description of this method.

Reviewer 2: To validate the transgenic genotypes, please show the following images:

Confocal images of YFP fluorescence in the Ai32/Rho-iCre and Ai32/HRGP-Cre should be added to Figure 1 or included as a supplementary figure.

Authors>> As requested, we added live cell images of YFP fluorescence illustrating the expression of channel rhodopsin 2 in rods and cones driven by Rho-iCre and HRGP-Cre, respectively (Fig 1, B-C).

Reviewer 2: Confocal images of Syt1 immunofluorescence in the OPL of wild type, rod ChR2/Syt1KO, cone ChR2/Syt1KO, and rod/cone ChR2/Syt1KO need to be added to Figure 3.

Confocal images of cx36 immunofluorescence in the OPL of wild type and Rod ChR2/Cx36KO need to be added to Figure 3.

Authors: >> Unfortunately, we no longer have mice lacking Syt1 in these various genotypes. It originally took 2 years to breed the proper Syt1 genotypes. After completing experiments on those mice, it took another year to obtain and breed the rod-specific Cx36KO mice. In an earlier series of studies, we confirmed selective elimination of Syt1 from rods and cones using the floxed Syt1 mouse line that we developed (Quadros et al., 2017; Grassmeyer et al., 2019; Mesnard et al., 2022; Sladek and Thoreson, 2023). These earlier studies included selective elimination of Syt1 from rods and/or cones in Ai32 mice. We genotyped all of the mice used in the present experiments and are therefore confident that Syt1 was successfully eliminated from rods and cones in the mice used for this study. We also note that green fluorescence from EYFP (shown in new Fig. 4) makes it difficult to detect changes in the green channel which in turn makes dual color IHC in these mice problematic.

Authors: >>While the ability of the floxed Cx36 gene to eliminate this protein in rods and cones using Rho-iCre and HRGP-Cre mice had been previously shown by Ribelayga and colleagues (Jin et al., 2020; 2022), we had not characterized this mouse line ourselves. We therefore followed the reviewer's suggestion that we confirm selective elimination of Cx36 in rods from Cx36fl/fl/Ai32/Rho-iCre mice. This effort took a few months to generate the mice needed for these experiments and refine IHC protocols. We added a new figure (Fig. 4) comparing Cx36 labeling in Ai32/Rho-iCre mice (expressing wild-type Cx36) and Ai32/Rho-iCre/Cx36flfl (rod-specific Cx36 KO). ChR2 in Ai32 mice is coupled to EYFP producing membrane-bound green fluorescence throughout the cell. We additionally labeled these retinas with an antibody for Syt1 using an Atto488-tagged nanobody. This produced additional strong labeling in the inner and outer plexiform layers superimposed on EYFP fluorescence in rods. To label Cx36, we used a rhodamine-conjugated secondary antibody. Rhodamine also labels blood vessels but in capillary-free regions, one can see punctate labeling of the Cx36 antibody in the OPL. Punctate labeling in the OPL was largely eliminated in rod-specific Cx36KO mice, although strong punctate labeling remained in the IPL. A few puncta were occasionally visible in the OPL, but as Jin et al. (2020) report, these are likely due to expression of Cx36 in cones. Elimination of puncta in the OPL is more clearly visible in the magnified image from the single confocal sections shown at the bottom of Fig. 4. Syt1 labeling did not appear to be changed in rod-specific Cx36KO mice.

Reviewer 2: The text referring to Figure 3 in the Results states "Optogenetic responses were absent in all 7 horizontal cells recorded in 3 cone-specific Syt1 knockout retinas" and "(Fig. 3C; 5 cells from 3 mice)" and "(Fig. 3D; n=8 cells from 3 mice)", but the current traces in Figs 3A, 3C, and 3D seem to be from only one cell each. Please make this clear in the figure legend.

Authors: >> We revised the text and figure legend to clarify that these are example responses.

Reviewer 2: Figure 3: The Results refers to the presence of spontaneous miniature post-synaptic currents in Fig 3A and 3C, but not 3D. Why not mention minis in 3D? What is the explanation for the apparent difference in the time course of the minis in 3A and 3D (the ones in 3A appear faster)? Is there an explanation for the slow decrease in the current in 3D? Authors: >> The bright blue light flash used to drive optogenetic responses of rods in Fig. 5D also drives endogenous light responses of blue-sensitive cones (as illustrated in Fig. 2C). The slow outward current in Fig. 3D is the endogenous cone-driven, light-evoked decrease in glutamate release. Along with eliminating transmission of optogenetic responses, eliminating Syt1 from cones eliminates their ability to transmit endogenous light responses and so this outward current is absent from Fig. 5A (cone-specific Syt1 KO).

Authors: >>We did not mention mEPSCs with reference to panels B and D since the endogenous light-evoked cone responses evident in these figures were sufficient to show the presence of intact synaptic connectivity. However, as suggested, we revised the text to include mention of mEPSCs in other genotypes, too. As we showed previously (Grassmeyer et al., 2019), eliminating Syt1 from cones eliminates Ca2+-dependent release but leaves Ca2+-independent spontaneous release unchanged. Individual Ca2+-independent release events that remain in Syt1 KO cones are more well-isolated from another than the more frequent Ca2+-dependent release events that can occur with intact Syt1.
